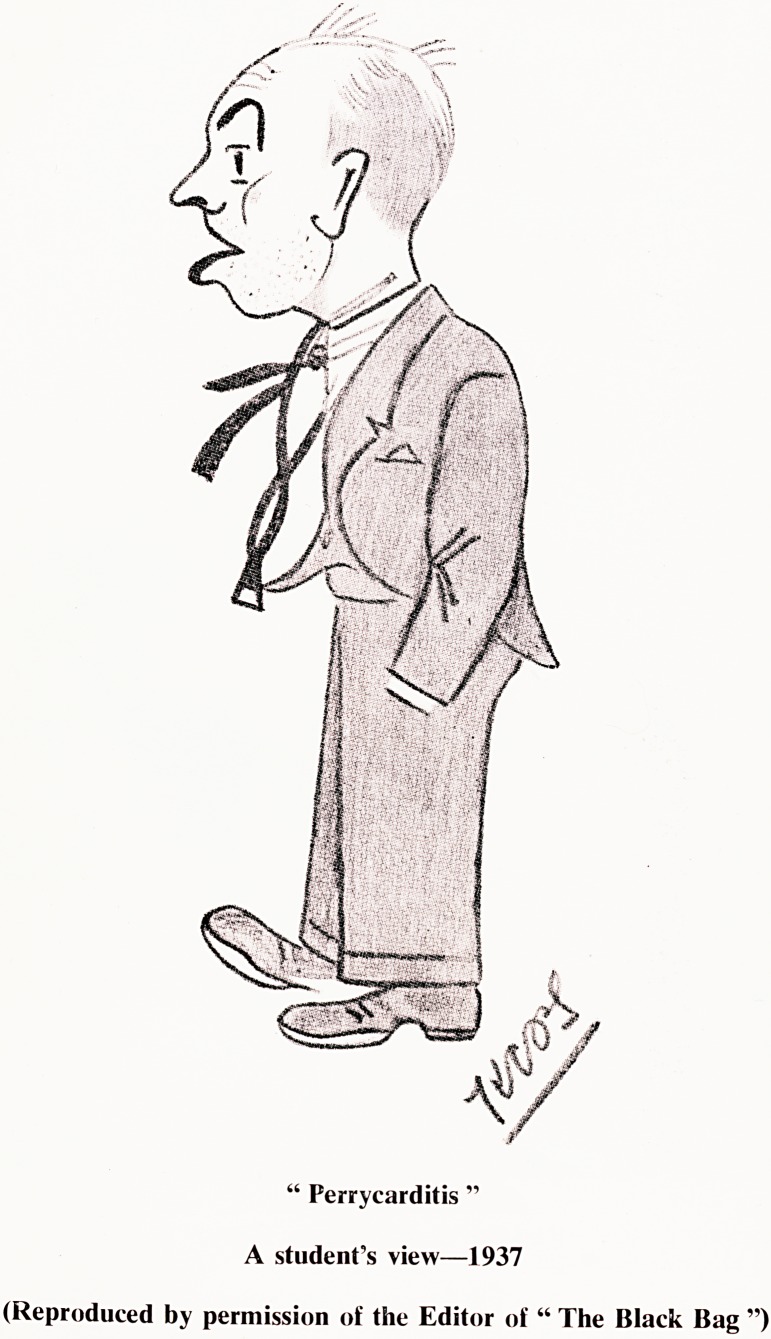# Editorial

**Published:** 1969-10

**Authors:** 


**Professor C. Bruce Perry**

**" Perrycarditis "**

**A student's view—1937**

**(Reproduced by permission of the Editor of " The Black Bag ")**

### EDITORIAL

### Professor C. Bruce Perry

On 31st July 1969 Professor C. B. Perry retired from the Chair of Medicine at the University of Bristol, after occupying it for no less than 34 years. It is difficult to comprehend what this means in terms of the influence this distinguished man has wielded over medical thought and practice in Bristol for more than a generation. Upwards of two thousand doctors throughout the world must have vivid memories of " Bruce's " rounds—so must the many thousands of nurses who have watched from a position of comparative safety the merciless grilling of one batch of raw students after another. How many mature practitioners must there be who have valued his advice in consultation? And what of the countless grateful patients who have come under his skilled and gentle care?

Born in 1903, Charles Bruce Perry was educated at Bristol Grammar School. Following a school tradition he crossed the road to study medicine at Bristol University, graduating M.B., Ch.B. with second class honours in 1926, winning also the Martyn Memorial prize, the Sanders Scholarship, and the gold medal for his year. After house appointments in Bristol and London he became research assistant, as a Colston research fellow, to the late Dr. Carey Coombs, thus laying the foundation of a life-long interest in cardiology. In 1928 he obtained both M.D. and M.R.C.P. and was appointed medical registrar to the Bristol General Hospital. In 1933 he became assistant physician there, with appointments also at Winford Hospital and the Royal Hospital for Sick Children and Women (as it then was).

In 1935 he become Bristol's first full-time Professor of Medicine. He brought to the teaching of medicine a refreshing clarity of mind and a critical approach which has persisted down the years. To many students at that time medicine seemed to be an almost totally vague and empirical subject and Perry's determined presentation of it as a logical discipline based on sound scientific observations had an immediate and lasting impact. While delighting in sweeping away mumbo-jumbo and exploding time-honoured myths, he nevertheless preserved and treasured all that was best in traditional British medicine. He insisted on sound learning and exact factual knowledge, and his reaction to sloppy thinking or attempts to evade the question could strike terror into his students. In time however they were to discover that behind the sometimes gruff and roaring exterior there was a most kind, sincere, and considerate man whose one desire was for their welfare and that of his patients.

He has served the University as Dean of the Faculty and from 1958 to 1961 was Pro-Vice-Chancellor. He gave the Long Fox Lecture in 1943.

Elected a Fellow of the Royal College of Physicians in 1936 he was Bradshaw lecturer in 1944 and Lumleian lecturer in 1969. He was a Censor of the College from 1962 to 1964. He has been President of the Association of Physicians of Great Britain and Chairman of the British Cardiac Society.

Always a strong supporter of the Bristol Medico-Chirurgical Society he was its President 1957-58 and his memorable presidential address on the subject of Medical Education is one of his several contributions to this Journal.

The papers to which this issue is devoted were delivered at a symposium of Medicine held in the Bristol Medical School on June 26th-28th, 1969, to honour Professor Bruce Perry on his retirement. It seemed appropriate in the last weeks of his hospital career to invite him to hear and criticize these papers.

In producing this special issue of the Journal in his honour we pay tribute to thirty-four years of loyal and devoted service to the teaching of medicine and wish him and Mrs. Perry many years of happy life in retirement.

### SYMPOSIUM IN HONOUR OF CHARLES BRUCE PERRY

**Professor of Medicine, University of Bristol, 1935-1969**

### CONTRIBUTORS

R. G. Charles, B.Sc. — Medical Student, University of Bristol.

J. A. Cosh, M.D., F.R.C.P. — Physician, Bath.

T. J. David — Medical Student, University of Bristol.

A. St. J. Dixon, M.D., F.R.C.P. — Rheumatologist, Bath.

G. R. Fearnley, M.D., F.R.C.P. — Physician, Gloucester.

J. Goodwin, M.D., F.R.C.P. — Professor, Royal Postgraduate Medical School, Hammersmith.

K. R. Gough, M.D., M.R.C.P. — Physician, Bath.

S. C. Jordan, M.D., M.R.C.P. — Cardiologist, Bristol.

J. C. Little, M.D., M.R.C.P., D.P.M. — Psychiatrist, Dumfries.

J. K. Lloyd, M.D., F.R.C.P., D.P.H. — Reader in Child Health, University of London.

H. G. Mather, M.D., F.R.C.P. — Physician, Bristol.

F. Nahai, B.Sc. — Medical Student, University of Bristol.

A. E. Read, M.D., F.R.C.P. — Professor Elect, University of Bristol.

Sir T. Holmes Sellors, D.M., M.Ch., F.R.C.S., F.R.C.P. — Surgeon, The Middlesex Hospital, London.

D. B. Shaw, M.D., M.R.C.P. — Physician, Exeter.

N. C. Tanner, M.D., F.R.C.S. — Surgeon, Charing Cross Hospital, London.

M. D. Turner, Ph.D., M.D., M.R.C.P. — Professor, University of Rochester, N.Y.

D. N. Walder, M.D., Ch.M., F.R.C.S. — Professor, University of Newcastle-upon-Tyne.

## Figures and Tables

**Figure f1:**
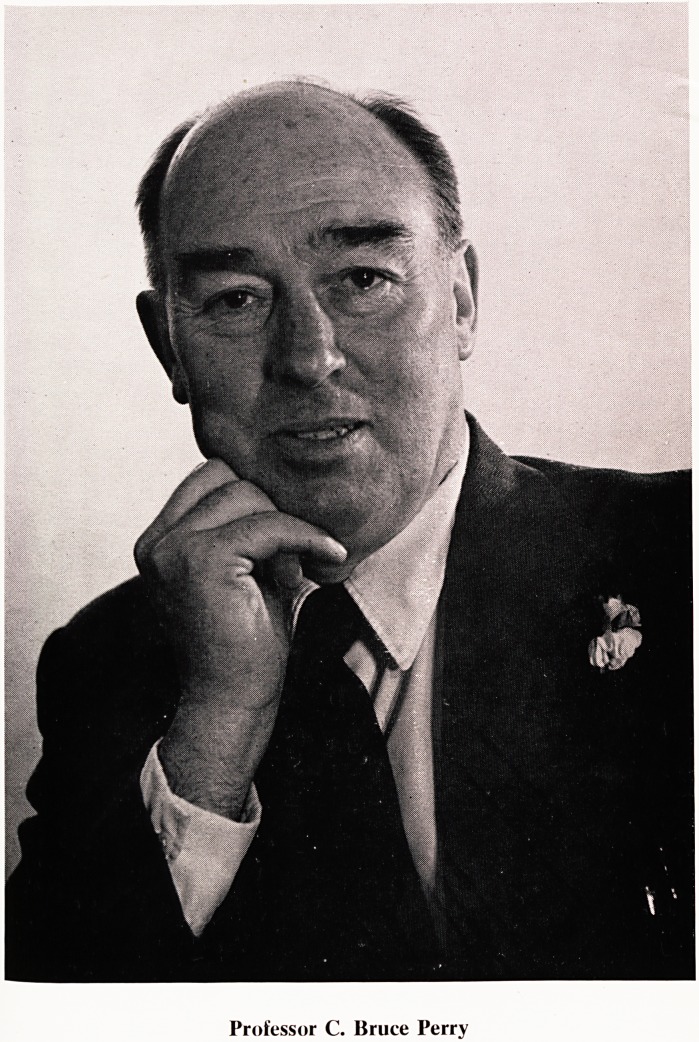


**Figure f2:**